# Endogenous Retroviruses Provide Protection Against Vaginal HSV-2 Disease

**DOI:** 10.3389/fimmu.2021.758721

**Published:** 2022-01-04

**Authors:** Radeesha Jayewickreme, Tianyang Mao, William Philbrick, Yong Kong, Rebecca S. Treger, Peiwen Lu, Tasfia Rakib, Huiping Dong, May Dang-Lawson, W. Austin Guild, Tatiana J. Lau, Akiko Iwasaki, Maria Tokuyama

**Affiliations:** ^1^ Department of Immunobiology, Yale University School of Medicine, New Haven, CT, United States; ^2^ Department of Internal Medicine, Section of Endocrinology, Yale School of Medicine, New Haven, CT, United States; ^3^ Department of Molecular Biophysics and Biochemistry, W.M. Keck Foundation Biotechnology Resource Laboratory, Yale University School of Medicine, New Haven, CT, United States; ^4^ Department of Microbiology and Immunology, Life Sciences Institute, The University of British Columbia, Vancouver, BC, Canada; ^5^ Howard Hughes Medical Institute, Chevy Chase, MD, United States

**Keywords:** HSV-2 (herpes simplex virus type-2), endogenous retroviruses (ERVs), sexually transmitted infections, antiviral response, vaginal infection

## Abstract

Endogenous retroviruses (ERVs) are genomic sequences that originated from retroviruses and are present in most eukaryotic genomes. Both beneficial and detrimental functions are attributed to ERVs, but whether ERVs contribute to antiviral immunity is not well understood. Here, we used herpes simplex virus type 2 (HSV-2) infection as a model and found that Toll-like receptor 7 (*Tlr7* ^-/-^) deficient mice that have high systemic levels of infectious ERVs are protected from intravaginal HSV-2 infection and disease, compared to wildtype C57BL/6 mice. We deleted the endogenous ecotropic murine leukemia virus (Emv2) locus on the *Tlr7* ^-/-^ background (*Emv2* ^-/-^ *Tlr7* ^-/-^) and found that *Emv2* ^-/-^ *Tlr7* ^-/-^ mice lose protection against HSV-2 infection. Intravaginal application of purified ERVs from *Tlr7^-/-^* mice prior to HSV-2 infection delays disease in both wildtype and highly susceptible interferon-alpha receptor-deficient (*Ifnar1^-^* ^/-^) mice. However, intravaginal ERV treatment did not protect *Emv2^-/-^Tlr7^-/-^* mice from HSV-2 disease, suggesting that the protective mechanism mediated by exogenous ERV treatment may differ from that of constitutively and systemically expressed ERVs in *Tlr7^-/-^* mice. We did not observe enhanced type I interferon (IFN-I) signaling in the vaginal tissues from Tlr7-/- mice, and instead found enrichment in genes associated with extracellular matrix organization. Together, our results revealed that constitutive and/or systemic expression of ERVs protect mice against vaginal HSV-2 infection and delay disease.

## Introduction

Genital herpes simplex virus 2 (HSV-2) is a common cause of sexually transmitted infection (STI) and affects 491.5 million individuals worldwide, equivalent to 13.2% of the world population ages 15 to 49 (1). A higher percentage of females are infected with HSV-2, but HSV-2 infection affects males as well (1). HSV-2 enters through epithelial cells of the skin and mucosal linings of the genital area and establishes a life-long infection by latently infecting the dorsal root ganglion neurons (2). Reactivation of HSV-2 occurs during stress and can cause blisters and lesions, itchiness, pain and shedding of the virus (2, 3). An estimated 60% of neonatal herpes infection through vertical transmission is fatal without treatment (4). In addition, epithelial cell damage caused by HSV-2 infection and reactivation results in STI coinfections, including human immunodeficiency virus (HIV) and hepatitis C virus (HCV) (3). There is also a higher incidence of bacterial vaginosis and encephalitis in HSV-2 positive individuals (5). Although acyclovir therapy effectively reduces the viral load of HSV-2, acyclovir resistant strains of HSV-2 have emerged (6), and there is no vaccine to prevent HSV-2 infection (6, 7). Thus, HSV-2 infection remains a public health concern.

Endogenous retroviruses (ERVs) are retroviral sequences that are part of most eukaryotic genomes and make up 8 to 10% of the human and mouse genomes, respectively (8, 9). Most ERV sequences are not full-length proviral sequences, and instead are solo-long terminal repeats (LTRs) that are important regulators of gene expression (10, 11). However, a fraction of ERV sequences in both humans and mice encodes proviral sequences that generate viral RNA and proteins (12, 13). Although infectious ERVs have not been reported in humans, human ERVs can generate viral-like particles (14, 15), and proviral ERVs in mice generate infectious ERVs (16, 17). There is mounting evidence that ERVs are dysregulated in various diseases, including cancer, autoimmunity, and during viral infection and contribute to the type I IFN (IFN-I) response (18). IFN-I induction associated with ERV dysregulation can reverse dampened immune responses in tumor cells and promote antitumor immunity (19). Similarly, IFN-I induction by ERVs may promote antiviral immunity, but this is not well understood.

ERV expression is tightly controlled, but suppression of innate and adaptive immunity results in elevated production of infectious ERVs in mice (16, 17). Infectious ERVs emerge in mice deficient in Toll-like receptor 7 (TLR7), and re-integration of these ERVs in the genome can cause leukemia in TLR3/7/9-deficient mice (17). Additional effects of infectious ERVs, including their potential impact on antiviral immunity, are not well understood. Therefore, we probed whether heightened expression of ERVs would mediate protection against exogenous viral infection and used HSV-2 as a model pathogen to test this hypothesis.

## Results

### *Tlr7* ^-/-^ Mice Are Resistant to HSV-2 Infection

**Figure 1:**
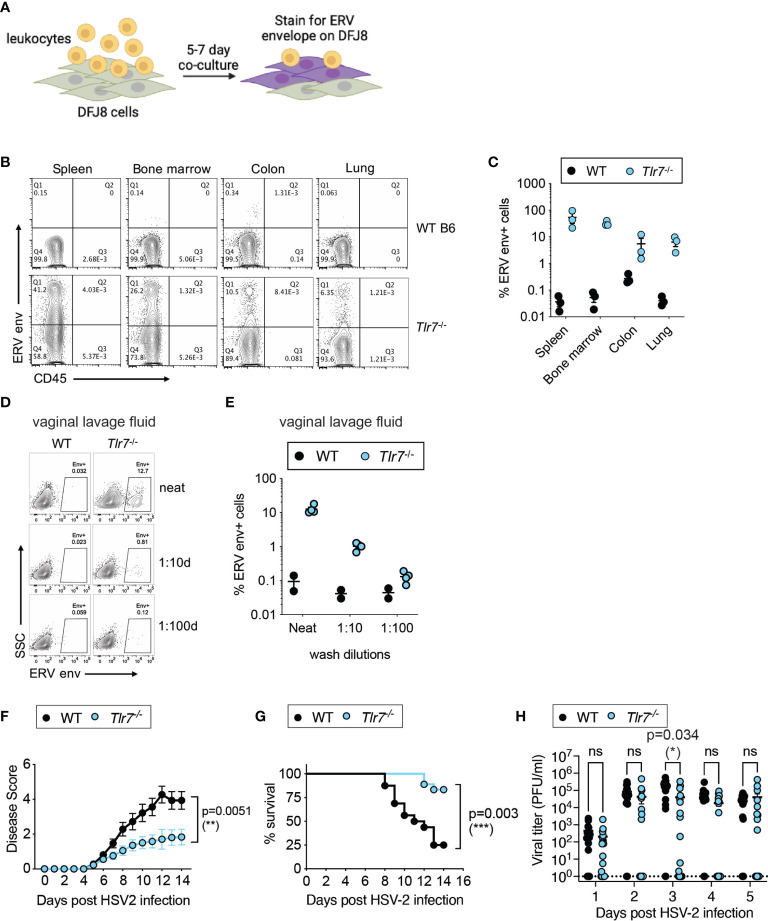
Elevated infectious ERVs and HSV-2 resistance in *Tlr7* ^-/-^ mice. **(A)** Schematic of DFJ8 co-culture assay to measure infectious ERVs. **(B, C)** Representative FACS plots of DFJ8 cells co-cultured with leukocytes from the indicated tissues for 7 days and stained with CD45 and ERV envelope, gated on live cells. Data from all mice were combined and plotted (n=3 per group). **(D, E)** DFJ8s cultured with dilutions of vaginal lavage fluid from WT B6 (n=2) or *Tlr7* ^-/-^ (n=4) and stained for ERV envelope protein. Representative data from more than three experiments. Mice were infected intravaginally with 2,500 PFU of HSV-2 and monitored for disease **(F)** and survival **(G)**, and vaginal viral titer **(H)** was quantified. The data are pooled from three independent experiments WT (n=16) and *Tlr7* ^-/-^ (n=18). Statistical significance was calculated using two-way ANOVA for disease score, Mantle-Cox test for survival and two-way ANOVA Sidak’s multiple comparisons test for viral titers. *p < 0.05; **p < 0.01; ***p < 0.001. ns, not significant.

We first quantified infectious ERVs in *Tlr7* ^-/-^ mice to determine whether ERVs are systemically elevated in *Tlr7* ^-/-^ mice. Total leukocytes from the spleen, bone marrow, colon and the lung were co-cultured with susceptible avian DFJ8 cells, and ERV envelope expression was measured on DFJ8 cells (**Figure 1A**). We observed that co-culturing of leukocytes from *Tlr7* ^-/-^ mice, but not wildtype (WT) mice, resulted in the expression of ERV envelope protein in DFJ8 cells, indicating that there was productive infection of DFJ8 cells by infectious ERVs (**Figures 1B, C**). In this assay, we excluded ERV envelope signal from any remaining input leukocytes in the culture by measuring ERV envelope expression in CD45^-^ DFJ8 cells. Similarly, vaginal lavage fluid from *Tlr7* ^-/-^ mice contained infectious virions (**Figures 1D, E**), indicating that ERVs are secreted into the vaginal lumen.

We next investigated whether systemic expression of infectious ERVs would influence antiviral responses and monitored outcomes to intravaginal HSV-2 infection in WT and *Tlr7* ^-/-^ mice. As HSV-2 is a DNA virus that is predominantly sensed by DNA sensors, innate sensing of HSV-2 should not be impaired in mice deficient in TLR7, an innate sensor for RNA ligands. Upon intravaginal HSV-2 infection, we observed a significant reduction in vaginal inflammation and disease pathology, as measured by disease score (**Figure 1F**). HSV-2 infected *Tlr7* ^-/-^ mice displayed local inflammation in the vagina, but infection did not cause hunching, hind/limb paralysis or lethality. *Tlr7* ^-/-^ mice infected with HSV-2 survived the infection compared to WT counterparts (**Figure 1G**), and this was accompanied by transient reduction in viral titers at 3 days post-infection (**Figure 1H**). Vaginal tissue staining for ERV envelope and HSV-2 showed HSV-2 replication in the superficial layer of the vaginal epithelium and in the *Tlr7* ^-/-^ tissue, HSV-2 was detected in epithelial cells where ERV envelope proteins were also highly expressed (**Supplementary Figure 1**). These data showed that *Tlr7* ^-/-^ mice with higher systemic expression of infectious ERVs are more resistant to intravaginal HSV-2 infection.

### Infectious ERVs From *Tlr7^-/-^* Mice Are Sufficient to Delay HSV-2 Pathology in WT Mice

**Figure 2:**
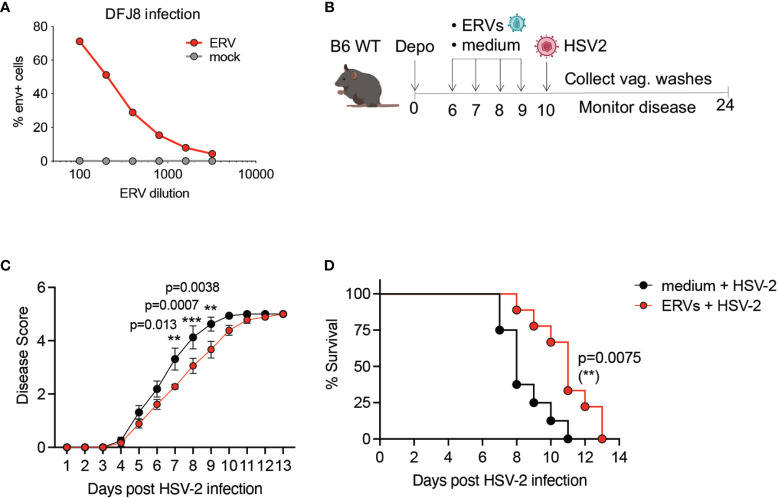
Purified ERVs from *Tlr7^-/-^* mice are sufficient to delay HSV-2 pathology in WT mice. **(A)** DFJ8 infection of purified ERVs compared to mock supernatant at the indicated dilutions measured by FACS using anti-ERV envelope antibody, gated on live cells. **(B)** Schematic of ERV pre-treatment followed by HSV-2 infection in WT B6 mice. Mice were pre-treated with purified ERVs (n=9) or medium (n=8) on the indicated days and infected intravaginally with 25,000 PFU of HSV-2 and monitored for disease **(C)** and survival **(D)**. The data are pooled from two independent experiments. Statistical significance was calculated using two-way ANOVA Sidak’s multiple comparisons test for disease score and Mantle-Cox test for survival. **p < 0.01; ***p < 0.001.

We next asked whether resistance to HSV-2 infection in *Tlr7* ^-/-^ can be recapitulated by ERVs alone. To test this, we amplified infectious ERV particles from *Tlr7* ^-/-^ mice, which we and others have previously shown is derived from a recombination between the Emv2 and Xmv43 ERV loci in C57BL/6 mice (16, 20). We used purified ERVs from *Tlr7* ^-/-^ mice that efficiently infected DFJ8 cells (**Figure 2A**) to determine whether exogenous ERV treatment would be sufficient to protect WT mice from HSV-2 infection. We intravaginally treated WT mice with either media or purified ERVs for four consecutive days prior to infection by HSV-2 (**Figure 2B**). Pre-treatment of mice with ERVs had no significant effect on pathology or vaginal HSV-2 titer in WT mice when infected at 2,500 PFU of HSV-2 (**Supplementary Figure 2**). However, when WT mice were infected with a much higher dose of HSV-2, 25,000 PFU, exogenous ERV pre-treatment was sufficient to delay pathology and death (**Figures 2C, D**). This effect was not a direct effect of ERVs on HSV-2, as incubation of HSV-2 with purified ERVs did not impact HSV-2 viral replication *in vitro* or infection *in vivo* (**Supplementary Figure 3**). Together, these data showed partial sufficiency of infectious ERVs in protection against HSV-2 infection.

### Emv2-Derived ERVs Are Necessary for Protection Against HSV-2

**Figure 3:**
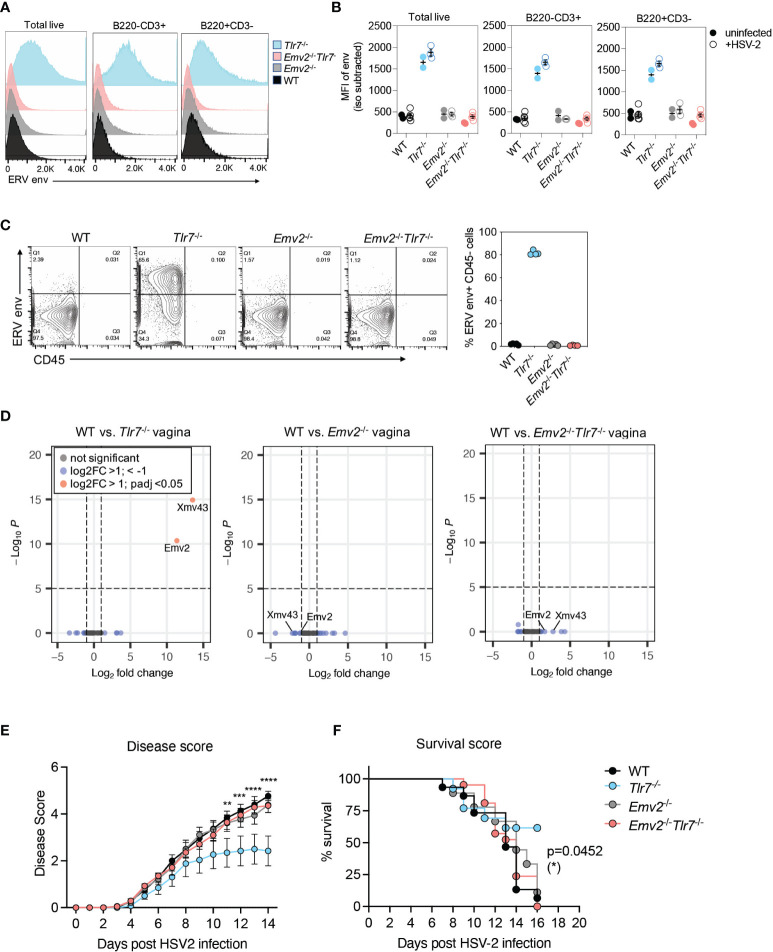
Emv2-encoded ERVs mediate protection against HSV-2. **(A)** Histograms of ERV envelope expression in the indicated splenocyte populations. Representative histogram of one mouse per genotype within an experiment with 3-5 mice per group. **(B)** ERV envelope expression data showing data from all mice and comparing splenocytes from uninfected mice (filled circles) with mice from 2 days post HSV-2 infection (open circles). **(C)** Quantification of ERV envelope expression on DFJ8 cells at 14 days post co-culture with splenocytes from the indicated mice (n=4 per group), as a proxy measurement for infectious ERVs. CD45^+^ input splenocytes were excluded to measure ERV envelope only in DFJ8 cells. **(D)** Differential proviral ERV expression in vaginal tissues from the indicated mice determined by RNA-seq analysis followed by DESeq2 analysis. Data are from 2 mice per group. Indicated mice were infected intravaginally with HSV-2 and disease score **(E)** and survival **(F)** were monitored (WT, n=15; *Emv2* ^-/-^, n=9; *Tlr7* ^-/-^, n=13; *Emv2* ^-/-^ *Tlr7* ^-/-^, n=21). The data are pooled from three independent experiments. Two-way ANOVA Tukey’s multiple comparisons test and log-rank (Mantel-Cox) test were performed to calculate significance for disease score and survival, respectively, between *Tlr7* ^-/-^ and *Emv2* ^-/-^ *Tlr7* ^-/-^ groups. *p < 0.05; **p < 0.01; ***p < 0.001; ****p < 0.0001.

The emergence of infectious ERVs in *Tlr7* ^-/-^ mice had previously been attributed to recombination between the single ecotropic ERV locus in C57BL/6 mice, Emv2 and Xmv43 (20, 21). We confirmed this by RNA-sequencing and observed that indeed *Tlr7* ^-/-^ mice expressed significantly higher levels of Emv2 (**Supplementary Figure 4**). To address whether ERVs were necessary for protection against HSV-2 in *Tlr7* ^-/-^ mice with elevated ERV expression, we deleted the entire Emv2 locus using CRISPR-Cas9 in C57BL/6N mice (**Supplementary Figure 5A**) and crossed them to *Tlr7* ^-/-^ mice (22) to obtain *Emv2* ^-/-^ *Tlr7* ^-/-^ mice. We confirmed full deletion of the Emv2 locus (Chr8:123425507-123434150, GRCm38/mm10) by Sanger sequencing (**Supplementary Figures 5B, C**). Compared to *Tlr7* ^-/-^ mice, these mice showed no expression of ERV envelope protein in splenocytes (**Figures 3A, B**) nor infectious ERVs, as measured by co-culturing splenocytes with DFJ8 cells (**Figure 3C**). We next confirmed the loss of Emv2 expression in both *Emv2* ^-/-^ and *Emv2* ^-/-^ *Tlr7* ^-/-^ mice by analyzing proviral ERV expression in RNA sequencing (RNA-seq) data of vaginal tissues obtained from naive mice (**Figure 3D**). *Emv2* ^-/-^ *Tlr7* ^-/-^ vagina had no expression of Emv2, and the expression of other ERVs including xenotropic (xmv), polytropic (pmv) and modified polytropic (mpmv) ERVs was neither elevated in *Tlr7* ^-/-^ vaginal tissue nor affected upon deletion of Emv2.

We next tested whether the loss of Emv2 in *Tlr7* ^-/-^ mice would result in a loss of protection against HSV-2. Compared to *Tlr7* ^-/-^ mice infected with HSV-2, *Emv2* ^-/-^ *Tlr7* ^-/-^ mice were not protected against HSV-2, and HSV-2 mediated pathology was comparable to that of WT mice (**Figure 3E**). Comparable survival was further observed between *Emv2* ^-/-^ *Tlr7* ^-/-^ and WT mice upon HSV-2 infection (**Figure 3F**). We observed comparable expression of HSV-2 entry receptors, Nectin-1 and HVEM (23, 24) across all genotypes, as well as between WT mice treated with media and mice treated with purified ERVs (**Supplementary Figure 6**). This suggests that receptor expression is not a determinant of differential susceptibility. These results revealed that the protection against HSV-2 in *Tlr7* ^-/-^ mice is dependent on elevated expression of infectious ERVs derived from Emv2 in *Tlr7* ^-/-^ mice.

Based on the observation that ERV treatment was sufficient to provide protection against HSV-2 in WT mice, we next tested whether exogenous ERV treatment could rescue the lack of ERVs in *Emv2^-/-^Tlr7^-/-^* mice. Both disease scores and survival were comparable between *Emv2^-/-^Tlr7^-/-^* mice treated with media alone or exogenously treated with ERVs, indicating that local treatment with ERVs is not sufficient to rescue the loss of protection observed in *Emv2^-/-^Tlr7^-/-^* mice. (**Supplementary Figure 7**).

### Transcriptome Analysis of Vaginal Tissues

**Figure 4:**
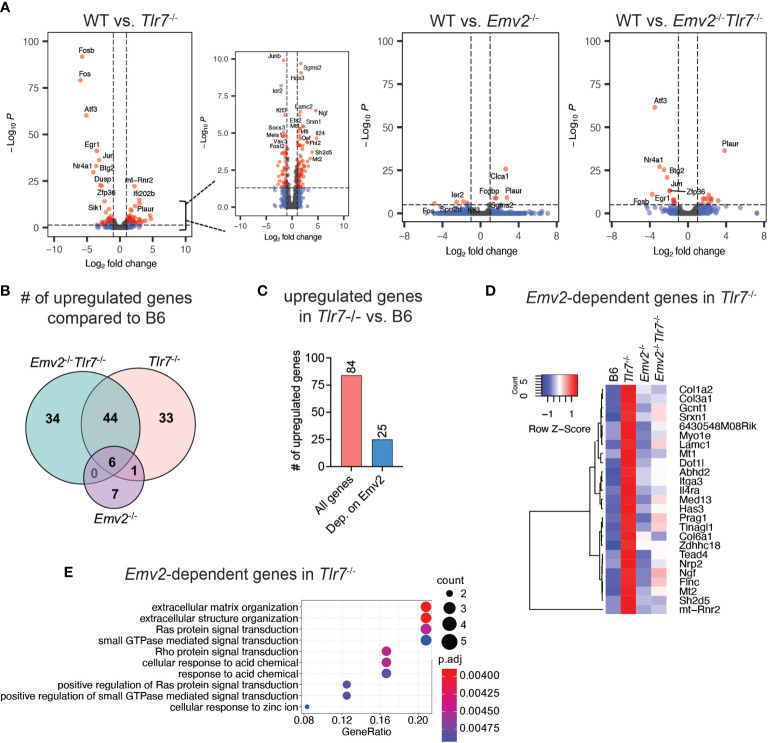
Transcriptome analysis of HSV-2 protected vaginal tissue. **(A)** Volcano plots of differentially expressed cellular genes between wildtype (WT) and *Tlr7* ^-/-^, *Emv2* ^-/-^, *Emv2* ^-/-^ *Tlr7* ^-/-^ vaginal tissues. Log2 fold change and p-adj (log10P) values were obtained by DESeq2. **(B)** Venn diagram depicting the number of significantly upregulated genes (log2FC > 1; padj < 0.05) in the indicated genotypes compared to WT. **(C)** Number of significantly upregulated genes in *Tlr7* ^-/-^ compared to WT and of those, number of genes that are not upregulated in *Emv2* ^-/-^ *Tlr7* ^-/-^. Heatmap **(D)** and gene enrichment analysis **(E)** of Emv2-dependent upregulated genes in*Tlr7* ^-/-^ mice.

In order to identify Emv2-dependent gene signatures in *Tlr7* ^-/-^ mice that correlate with protection against HSV-2, we performed cellular transcriptome analysis on whole vaginal tissues obtained from Depo-treated naive WT, *Tlr7* ^-/-^, *Emv2* ^-/-^ *Tlr7* ^-/-^ and *Emv2* ^-/-^ mice. We analyzed differential expression of genes (DEG) and compared the cellular transcriptome between WT vaginal tissue and *Tlr7* ^-/-^, *Emv2* ^-/-^, or *Emv2* ^-/-^ *Tlr7* ^-/-^ vaginal tissues (**Figure 4A**). More than half of the genes upregulated in *Tlr7* ^-/-^ tissue overlapped with those upregulated in *Emv2* ^-/-^ *Tlr7* ^-/-^ tissues (**Figure 4B**), but we found 25 genes that were significantly upregulated in *Tlr7^-/-^* tissue compared to *Emv2^-/-^Tlr7^-/-^* tissue (**Figure 4C**). These genes likely represent an Emv2-dependent gene signature that is associated with HSV-2 protection in *Tlr7* ^-/-^ vaginal tissues (**Figures 4C, D**). We further performed gene enrichment analysis for this set of genes and found that the most significantly enriched cellular pathway that is represented by this gene set was extracellular matrix organization (**Figure 4E**). Notably, these genes were devoid of interferons and interferon-stimulated genes. Taken together these data revealed a unique set of genes that are induced in HSV-2 protected *Tlr7* ^-/-^ mice that are regulated by infectious ERVs.

### Type I IFN Does Not Play a Major Role in ERV-Mediated Protection Against HSV-2

Type I IFN (IFN-I) is a key mediator of innate antiviral immunity and signals through the IFNα/β receptors to induce expression of IFN-stimulated genes (ISGs) (25). IFN-I protects against intravaginal HSV-2 infection in mice by initiating a protective innate and adaptive immune response (26). In parallel, dysregulation of ERVs is associated with the induction of an IFN-I response through RIG-I/MDA5 (19). Thus, we originally hypothesized that antiviral response against HSV-2 occurs through ERV-mediated induction of IFN-I signaling. However, based on the transcriptome analysis, IFN signaling was not one of the enriched pathways in *Tlr7* ^-/-^ vaginal tissue (**Figure 4E**). Thus, we further probed whether IFN-I signaling was involved in ERV-mediated protection against HSV-2.

**Figure 5:**
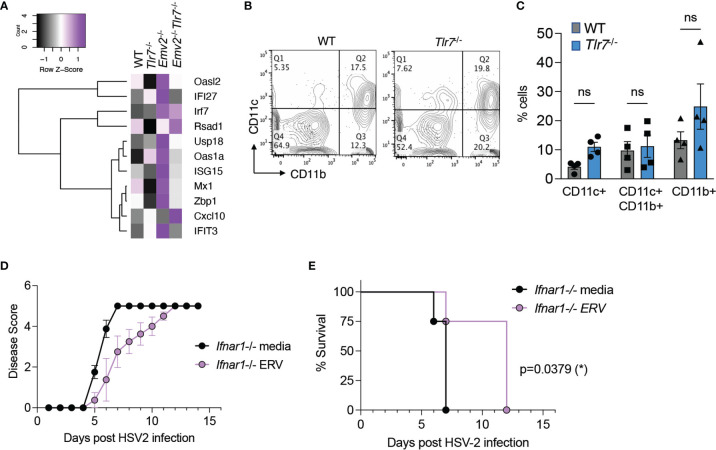
Purified ERVs from *Tlr7^-/-^* mice confer protection against HSV-2 disease in the absence of IFN-α/β receptor. **(A)** Heatmap depicting expression levels of IFN-stimulated genes in the indicated vaginal tissues. **(B)** A representative flow cytometry plot depicting CD11b and CD11c staining on vaginal tissues from naïve WT and *Tlr7* ^-/-^ mice, gated on singlets/live/CD45+ cells. **(C)** Compiled data for flow cytometry analysis of CD11b^+^ and CD11c^+^ cells (n=4 per group). Two-way ANOVA with Sidak’s multiple comparisons were performed to calculate statistical significance. ns, not significant. **(D, E)** HSV-2 infection of *Ifnar1^-/-^* mice treated intravaginally with optiMEM (media) or ERVs (n=4 mice per group) and monitored for disease score and survival. Log-rank (Mantel-Cox) test was performed to calculate significance. *p < 0.05.

We examined expression of a set of ISGs that has previously been associated with protection against intravaginal HSV-2 infection (27). Based on RNA-seq data, these ISGs were not significantly induced in *Tlr7* ^-/-^ vaginal tissues compared to WT vagina (**Figure 5A**). We also examined whether CD11c^+^ dendritic cells that are responsible for mounting a protective IFN-I response in the vagina were elevated in *Tlr7* ^-/-^ mice. We found comparable levels of live CD45^+^ cells (**Supplementary Figure 8**) as well as CD11c^+^ and CD11c^+^CD11b^+^ dendritic cells in the vagina of WT and *Tlr7* ^-/-^ mice (**Figures 5B, C**). Finally, we tested whether protection against HSV-2 by exogenous ERV treatment requires IFN-I signaling by treating *Ifnar1^-^* ^/-^ mice intravaginally with purified ERVs from *Tlr7^-/-^* prior to HSV-2 infection. We observed that exogenous ERV treatment is capable of delaying disease progression both by disease score and survival in *Ifnar1^-^* ^/-^mice (**Figures 5D, E**), indicating that IFN-I signaling is not required for this protection. The lack of ISG expression in mice that are protected from HSV-2, together with the observation that protection is provided by ERVs even in the absence of IFN-I signaling, suggest that IFN-I is not a major protective mechanism conferred by Emv2-derived ERVs in *Tlr7* ^-/-^ mice or by purified ERVs from *Tlr7* ^-/-^ mice.

## Discussion

ERVs are genomic sequences that originated from retroviral infections and make up a significant proportion of most eukaryotic genomes (9). In humans, ERVs can generate viral-like particles but infectious ERVs have not been detected. In mice, ERVs are grouped as ecotropic, polytropic, and modified polytropic murine leukemia viruses (MLVs), originally studied for their oncogenic properties, similar to exogenous MLVs such as Moloney, Rauscher, Abelson and Friend MLVs (28). Although the propensity for individual ERV loci to generate infectious viral particles is low, recombination between sequences from distinct ERV loci can result in the generation of replication competent retroviruses, which can be amplified under immunosuppressive conditions. Beyond the capacity to produce infectious retroviruses, ERV sequences encode viral proteins that affect both the innate and adaptive immune responses and are potential sources of cytosolic ligands for innate immune stimulation (18). Although ERVs are dysregulated during viral infection in humans, the potential for ERVs to impact antiviral immunity is not well understood. Here we investigated whether ERVs affect antiviral responses and found that ERVs derived from the Emv2 locus of B6 mice confer protection against intravaginal HSV-2 infection *in vivo*.

The exact mechanism of ERV-mediated protection against HSV-2 remains unknown. We observed a modest and transient reduction in HSV-2 viral titers in the vaginal wash of *Tlr7* ^-/-^ mice compared to wildtype mice. This is consistent with a previous report showing that transient and early reduction in HSV-2 titers in the vaginal wash of mice treated with antibiotics result in significant improvement in disease scores (27). However, further examination is needed to determine whether the viral titer in the dorsal root ganglion where HSV-2 establishes latency is reduced. It also needs to be determined whether the ERV-mediated protection requires infectious ERVs in order to reveal the molecular mechanism of protection. Based on observations that ERVs are not directly acting on HSV-2 itself, ERVs are likely modulating the host to promote an antiviral state in the vaginal tissue. In line with this, transcriptome analysis revealed that extracellular matrix organization and Ras signaling pathways are enriched in *Tlr7* ^-/-^ vaginal tissues compared to WT and depend on the presence of Emv2. Extracellular matrix proteins including collagen and laminin form the structural basis of the dermal layer of the mucosal epithelium and support the physical barrier provided by the epithelium (29). Therefore, elevated expression of genes involved in extracellular matrix organization in *Tlr7* ^-/-^ vaginal tissues may broadly enhance vaginal epithelial integrity, which in turn confer protection against HSV-2. Future investigation will explore this and other pathways that may be involved in the protective response.

We observed a limited protective effect of ERV treatment in WT mice compared to the protection observed in *Tlr7^-/-^* mice. In addition, intravaginal treatment by purified ERVs was not sufficient to rescue the loss of protection in *Emv2-/-Tlr7^-/-^* mice. There are several potential reasons for these observations. One possibility is that the duration and amount of ERVs expressed in *Tlr7^-/-^* mice are much higher than the amount of exogenous virus provided through intravaginal administration. Constitutive production of infectious ERVs in *Tlr7^-/-^* mice likely has a much broader impact on the vaginal tissue, as shown by the transcriptome differences (**Figure 4**). Another possibility is that TLR7 signaling is required for the partial protection provided by exogenous ERV treatment. It is also possible that expression of ERVs in the periphery or in other tissues is required to provide robust protection against HSV-2. Future studies will test these possibilities and dissect the signaling requirements for HSV-2 protection.

Studies have shown that activation of TLR7 signaling by TLR7 agonists confers protection against genital HSV-2 infection in guinea pigs and in humans (30, 31). This is in line with the potent antiviral effect of TLR signaling and innate immune activation. We initiated our study in TLR7-deficient mice because these mice, in particular, were reported to have high levels of ERVs (16, 17). However, to delineate the functional role of ERVs from the role of TLR7 signaling in antiviral immunity, we generated an Emv2-deficient mouse on the *Tlr7* ^-/-^ background and observed a loss of protection against HSV-2. Thus, the data support our conclusion that resistance to HSV-2 disease in *Tlr7^-/-^* mice requires infectious ERVs.

Intravaginal treatment of WT mice with purified ERVs from *Tlr7^-/-^* mice delayed disease progression and prolonged survival, but the effect was modest. This may suggest that the predominant effect of ERVs is to boost early antiviral response and reduce viral replication, while having a lesser impact on T cell immunity that provides robust protection throughout the course of infection (32). It is well established that HSV-2 infects the dorsal root ganglion (DRG) upon vaginal infection to establish latency, and immune restriction of DRG infection confers protection against HSV-2 (2). There remains the possibility that ERV-mediated protection against HSV-2 and delay in disease result from reduced HSV-2 infection of the DRG. Although HSV-2 establishes latency upon vaginal infection in mice, unlike human HSV-2 infection, latent HSV-2 does not reactivate. Our study is therefore limited to understanding the protective role of ERVs during the acute phase of vaginal HSV-2 infection in mice. Future studies will investigate the role of ERVs in different stages of HSV-2 pathogenesis.

Elevated expression of transposable elements including ERVs and LTR elements can coincide with IFN-I induction by cytosolic sensing of double stranded RNA and DNA (19, 33). In these settings, there are no infectious ERVs, but rather a dysregulation of retroelements that results in an IFN response that depends on cytosolic sensors, RIG-I/MDA5 and cGAS. ERVs from *Tlr7^-/-^* mice, however, are replication competent and consequently will likely stimulate innate sensors that recognize viral RNA during viral entry and replication. As generation of double-stranded RNA and DNA species are not part of the retroviral life cycle, sensing of infectious ERVs is expected to be distinct from cytosolic sensing of retroelements. The lack of IFN-I induction by heightened ERV expression in *Tlr7* ^-/-^ mice may in part be due to the lack of TLR7, which is a known innate sensor for retroviral infection necessary for viral control (34, 35). Although it remains to be determined whether exogenous ERV treatment induces an IFN-I response in wildtype vaginal tissue, a previous study showed that infectious ERVs do not readily induce an IFN-I response in wildtype mice, even in the presence of functional TLR7 signaling (17). In the absence of a robust IFN-I response however, our study revealed a potentially IFN-independent mode of HSV-2 protection, and elucidation of molecular mechanisms underlying these findings has the potential to reveal novel insights into antiviral responses to HSV-2.

## Materials and Methods

### Mice

C57BL/6N mice (strain 027) were obtained from Charles River Laboratories and bred in our animal facility. *Tlr7* ^-/-^ mice were bred in our animal facility (22). All mice were housed under specific-pathogen-free conditions and cared for according to Yale University IACUC guidelines. All female mice were used at eight to ten weeks of age for experiments.

The Emv2 knockout mouse model was generated by CRISPR-Cas9 methodology as described (36). In brief, T7-sgRNA templates were prepared by PCR, incorporating the guide sequences from the desired target regions in the mouse Emv2 locus (Mus musculus strain C57BL/6J, chr8:123425507-123434150, GRCm38/mm10), with a 5’ guide sequence of AGATTTAAGAGGAACAGCGC (sense orientation) and a 3’ guide sequence of CACAAGTCATCAGAATCGTC (antisense orientation). The T7-sgRNA PCR templates were then used for *in vitro* transcription and purification with the MEGAshortscript T7 Transcription Kit and MEGAclear Transcription Clean-Up Kit, respectively (both from Thermo Fisher Scientific). Cas9 mRNA (CleanCap, 5-methoxyuridine-modified) was purchased from TriLink Biotechnologies. Cytoplasmic microinjections of sgRNAs (at 50 ng/ul each) and Cas9 mRNA (at 100 ng/ul) into single-cell embryos at 0.5 d pc were performed by the Yale Immunobiology CRISPR Core. We have not performed whole-genome sequencing on these mice to exclude possible off-target gene editing.

Deletion of the *Emv2* locus was confirmed by sequencing the inserts amplified using the following primers (Emv2KO primers):

Fwd: AACCGGACCCCACTCAAAG

Rev: GCATAGAAAGGGGTTAAGAAATCC

The same primer sets were used to detect a knock-out band and the following primers used to detect the wild-type allele (Emv2WT primers):

Fwd: CCAGCTTGGGGGTCTTTCAAG

Rev: CGGACCCCACTCAAAGGC

A single line of confirmed Emv2 knockout mouse was backcrossed to B6N mice, and the heterozygous mice were crossed to generate a homozygous Emv2 knockout strain (*Emv2* ^-/-^). *Emv2* ^-/-^ mice were then crossed to *Tlr7* ^-/-^ mice to generate *Emv2* ^-/-^ *Tlr7* ^-/-^ mice. The following primers were used for *Tlr7*:

WT Fwd: AGGGTATGCCGCCAAATCTAAAG

Rev: ACCTTTGTGTGCTCCTGGAC

KO Fwd: TCATTCTCAGTATTGTTTTGCC

### HSV-2 Infection

Mice were injected subcutaneously in the neck scruff with 2mg per mouse of medroxyprogestrone acetate (Depo-Provera; GE Healthcare) six days prior to HSV-2 infection or ERV treatment. This treatment syncs mice in diestrus phase for up to 30 days post-administration and enhances HSV-2 susceptibility (37). On the day of infection, vaginal tract of mice was swabbed with a phosphate buffer saline (PBS)-soaked calcium alginate swab (Puritan Medical Products) and infected intravaginally with 10ul of 2500 PFU of wild-type (WT) HSV-2 strain (186syn+) (Spang, Knipe 1983 JVI). Infected mice were weighed and scored daily for two weeks for pathology based on scoring criteria that monitor local inflammation and physical pathology. Disease scores were obtained as follows: (0) no inflammation, (1) genital inflammation, (2) genital lesions and hair loss, (3) hunched posture and ruffled fur, (4) hind-limb paralysis or pre-moribound and (5) euthanized. Mice were euthanized before reaching a moribund state. In our experiments, infection of wildtype B6 mice with 2500PFU of HSV-2 186 Syn+ strain results in 80-100% of mice succumbing to infection and requiring euthanasia by 8 to 14 days post-infection.

Vaginal washes were collected daily for the first five days post-infection by collecting vaginal swabs and washes with 50ul of sterile PBS diluted 1:20 in buffer (PBS, 0.5mM MgCl2.6H2O, 0.9mM CaCl2.2H2O, 1% FBS, 1% Glucose). Washes were used to titrate HSV-2 on Vero cells (CCL-81; ATCC). WT HSV-2 was a generous gift from D. Knipe (Harvard Medical School) and was propagated on Vero cells as previously described (38). All animal procedures were performed in compliance with Yale Institutional Animal Care and Use Committee protocols.

### Tissue Harvest and Single Cell Preparation

Mouse vagina was harvested and processed as previously described to obtain single cell suspensions. Briefly, vaginal tissues were minced and digested in 1.65mg/ml Dispase II (Sigma) for 15 minutes in a 37°C shaking water bath, washed in PBS, and digested in 0.425mg/ml Collagenase D (Sigma), 30ug/ml DNase (Roche), 100u/ml Hyaluronidase (Sigma) in complete DMEM (10% FBS, 1% penicillin-streptomycin, 1% HEPES, 1% sodium pyruvate, 2-mercaptoethanol) for 30 minutes in a 37°C shaking water bath. Cells were passed through a 70um cell strainer and washed in PBS to obtain a single cell suspension. Splenocytes and bone marrow leukocytes were obtained as previously described (38). Leukocytes from the lung were obtained by tissue digestion as previously described (39). Lamina propria leukocytes were obtained from the small intestine of mice by removing the small intestine (SI) from the cecum and processed as previously described (40). Briefly, SI was cut longitudinally and washed in cold PBS three times to remove feces. Each SI was cut into three to four pieces and digested in 5mM EDTA in Hank’s Balanced Salt Solution (HBSS, Thermo Fisher) for 20 minutes in a 37°C shaking water bath. SI were then minced and digested in HBSS containing 2% FBS, 0.04mg/ml of DNase I (Roche), 4uM beta-mercaptoethanol, 1mg/ml of collagenase VIII (Sigma) for 45 minutes at 37°C shaking water bath. Cells were passed through a 100um cell strainer and washed in PBS to obtain a single cell suspension.

### Quantification and Propagation of Infectious ERVs

Infectious ERVs were quantified using a co-culture system using DFJ8 avian fibroblast cell line (kindly provided by Walther Mothes, Yale University). DFJ8 cells were co-cultured with single cell suspension of cells at a ratio of 50:1 lymphocytes to DFJ8 ratio in a 12-well plate for four days. Cells and supernatants were then transferred into a 60mm^2^ dish for three more days, and on the seventh day, cells are harvested and stained for mouse CD45 and MLV envelope antibody clone 573 (kindly provided by Leonard Evans, NIH) (41). CD45 cells were excluded and MLV envelope expression is quantified on DFJ8 cells.

Infectious ERVs from *Tlr7* ^-/-^ mice were generated from a single cell colony of DFJ8 cells co-cultured with *Tlr7* ^-/-^ splenocytes, as described previously (20). These DFJ8s stably express high levels of ERVs and were used to amplify ERVs. ERVs were harvested from the supernatant of DFJ8s cultured for seven days. Cells were removed by centrifugation and filtration of the supernatant through a 0.45um filter. Supernatant was concentrated through ultracentrifugation for two hours at 23,000 x g over 25% sucrose, and the pellet was resuspended in optiMEM media.

### Flow Cytometry

ERV envelope on lymphocytes was detected as previously described (42; b)using an anti-MLV envelope antibody clone 83A25 (kindly provided by Leonard Evans, NIH) (41). The following antibodies were used for staining of vaginal dendritic cells: Fixable Aqua Dead Cell Stain Kit (Thermo Fisher), CD45 (clone 30-F11, BioLegend), CD11b (clone M1/70, BioLegend), and CD11c (clone N418, BioLegend). All cells were stained in 1% BSA PBS and incubated on ice for 15 to 20 minutes. Cells were acquired on BD LSRII cytometer and analyzed by FlowJo software v8.8.7 (Tree Star, Inc.).

### RNA-Seq of Vaginal Tissues

Vaginal tissue was harvested, flash frozen, and placed in RLT lysis buffer (Qiagen RNeasy Kit) in bead homogenizer tubes (MP Biomedicals). The tissue was homogenized for one minute at maximum speed on a tissue homogenizer, and the lysate was clarified twice by centrifugation at 13,000rpm for 1 minute. RNA was purified from the clarified lysates according to manufacturer’s instructions (RNeasy Kit, Qiagen). 0.5ug of purified RNA was used to prepare a library using NEB sequencing kit (NEBNext Ultra II RNA Library Prep Kit for Illumina sequencing, NEB) according to instructions and sequenced on Illumina NextSeq 550 using 150bp pair-end sequencing using a high throughput flow cell. RNA-seq data was analyzed for ERVs and cellular data as previously described (42). The raw data files have been deposited in GSE185281.

### Confocal Microscopy

Vaginal tissues were harvested, placed in optimal cutting temperature (O.C.T.) compound (Fisher Scientific), flash frozen in dry ice, and stored at -80°C. Frozen tissues were sectioned into 7 mm thick sections and placed onto Superfrost microscope glass slides (Fisher Scientific). Sections were fixed in ice-cold acetone for 10 minutes at room temperature (RT), air dried, and blocked in blocking buffer (0.1M Tris-HCl pH7.4, 1% FBS, 2% goat serum) for 1 hour at RT. Sections were stained with anti-ERV envelope (83A25, 100mg/ml) and CD45.2 biotin (BioLegend, 10mg/ml) in staining buffer (0.1M Tris-HCl, 1% FBS) for 1 hour at RT and washed three times for 5 minutes in wash buffer (0.1M Tris-HCl, pH7.4). Then stained with HSV-2 gD FITC (ViroStat, 5mg/ml), anti-rat IgG Cy3 (Jackson Immuno, 40mg/ml), and streptavidin APC (BioLegend, 40mg/ml) in staining buffer for 1 hour at RT and washed three times. Sections were stained with Hoechst 33342 (ThemoFisher) at 1:100 for 10 minutes at RT, washed once in wash buffer, and ProLong Gold Antifade Mountant (ThermoFisher) was added. Z-section images were obtained on a Leica SP8 confocal microscope using a 40x oil immersion objective.

### Statistical Analysis

Statistical analyses were performed in Graphpad Prism9 software and the statistical tests used for each dataset is indicated in the figure legends. Differential gene expression was calculated using DESeq2 (43) using Bioconductor R (44). Heatmaps and volcano plots were generated using ggplot2 packages (45) and gene enrichment analysis was performed using clusterProfiler package (46) in Bioconductor R.

## Data Availability Statement

The datasets presented in this study can be found in online repositories. The names of the repository/repositories and accession number(s) can be found below: https://www.ncbi.nlm.nih.gov/geo/, GSE185281.

## Ethics Statement

The animal study was reviewed and approved by Yale University IACUC.

## Author Contributions

RJ and TM designed, performed and analyzed data; WP generated Emv2-/- mice; YK analyzed sequencing data; PL performed and analyzed data; HD and TR maintained and genotyped mice; RT generated key resource; MDL, WAG, and TJL performed experiments; AI wrote the manuscript, designed experiments, and edited final manuscript; MT wrote the manuscript, designed and performed experiments, analyzed data, and edited final manuscript. All authors contributed to the article and approved the submitted version.

## Funding

This work was supported in part by Howard Hughes Medical Institute (to AI) and by NIH awards R01AI127429 and R01EB000487 (to AI), NIH T32 AI89704-3 (to MT), T32 AI007019 (to TM), T32 GM00720540 and F30 AI 129265 (to RT). RJ was supported through the Yale College First-Year Summer Research Fellowship in the Sciences and Engineering. TR was supported through AbbVie Funding.

## Conflict of Interest

AI reported grants from AbbVie during the conduct of the study; “other” from RIGImmune; and personal fees from Boehringer Ingelheim outside the submitted work.

The remaining authors declare that the research was conducted in the absence of any commercial or financial relationships that could be construed as a potential conflict of interest.

The reviewer DK has declared a shared affiliation with one of the authors RT to the editor at the time of review.

## Publisher’s Note

All claims expressed in this article are solely those of the authors and do not necessarily represent those of their affiliated organizations, or those of the publisher, the editors and the reviewers. Any product that may be evaluated in this article, or claim that may be made by its manufacturer, is not guaranteed or endorsed by the publisher.
