# Supplementary figures and images for: Endogenous Retroviruses Provide Protection Against Vaginal HSV-2 Disease
Source: Front Immunol. 2022 Jan 4;12:758721. doi: 10.3389/fimmu.2021.758721 (PMC8764156; doi:10.3389/fimmu.2021.758721)

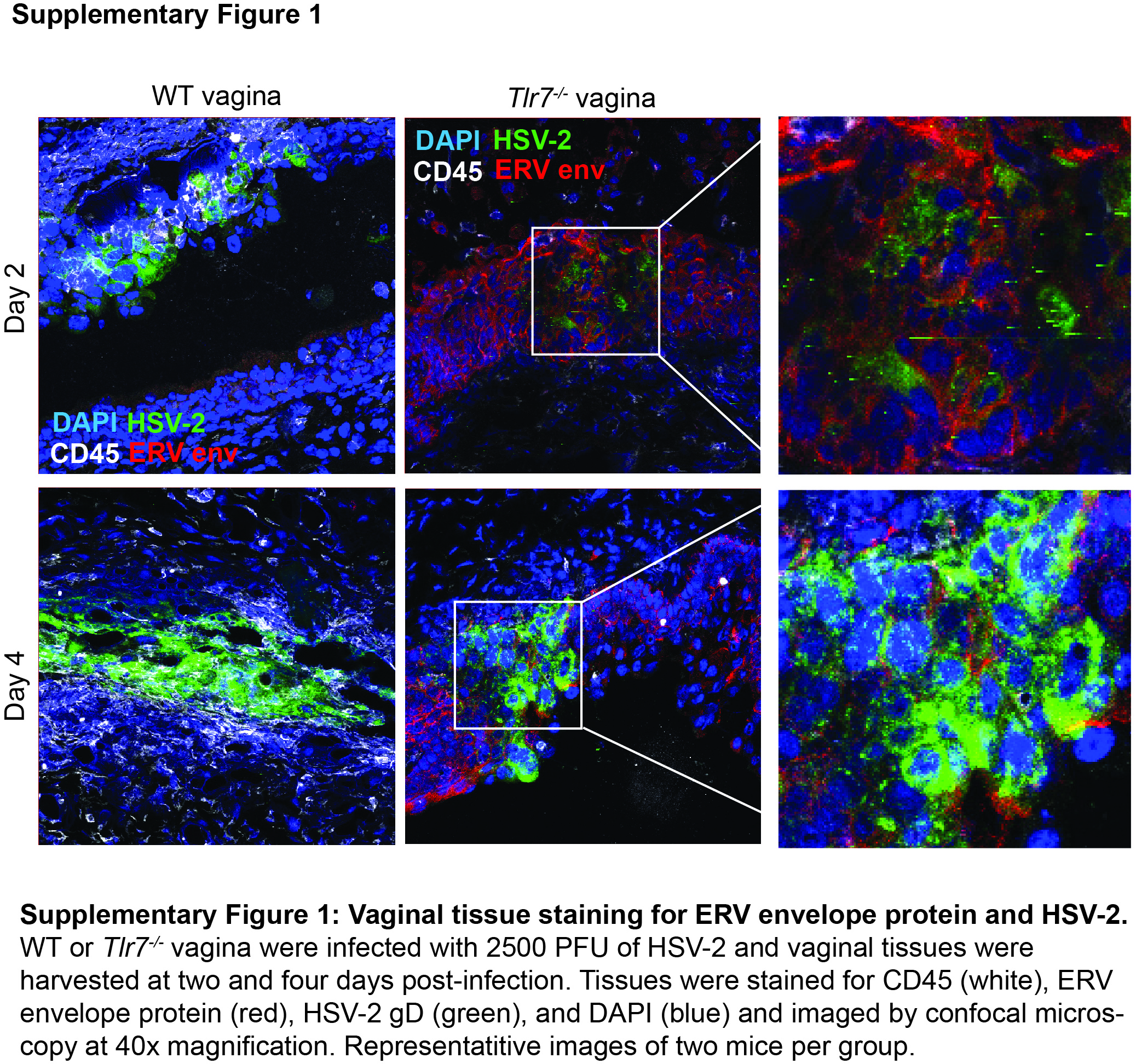

Supplement: Supplementary file 1 [file Image_1.jpeg]

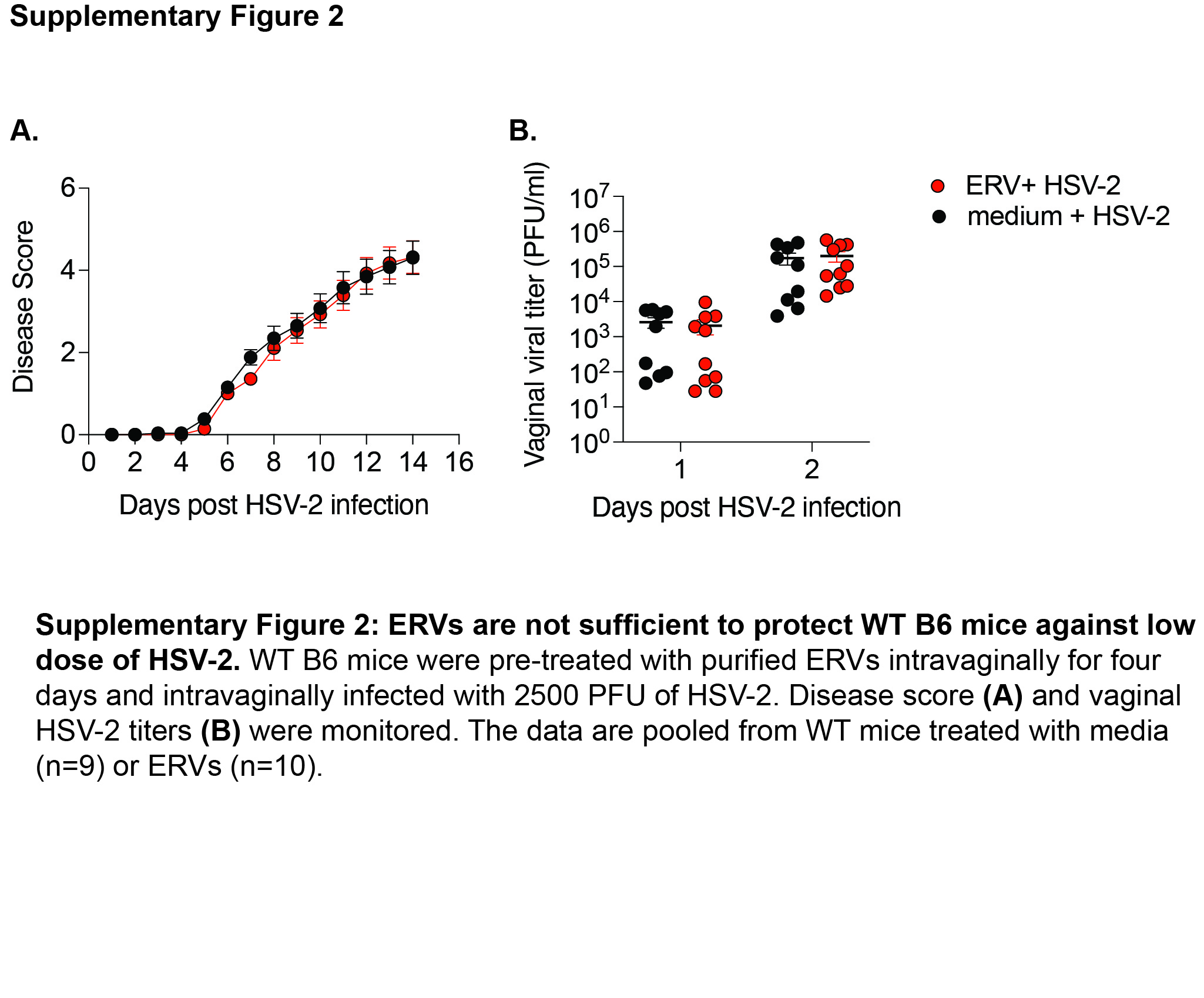

Supplement: Supplementary file 2 [file Image_2.jpg]

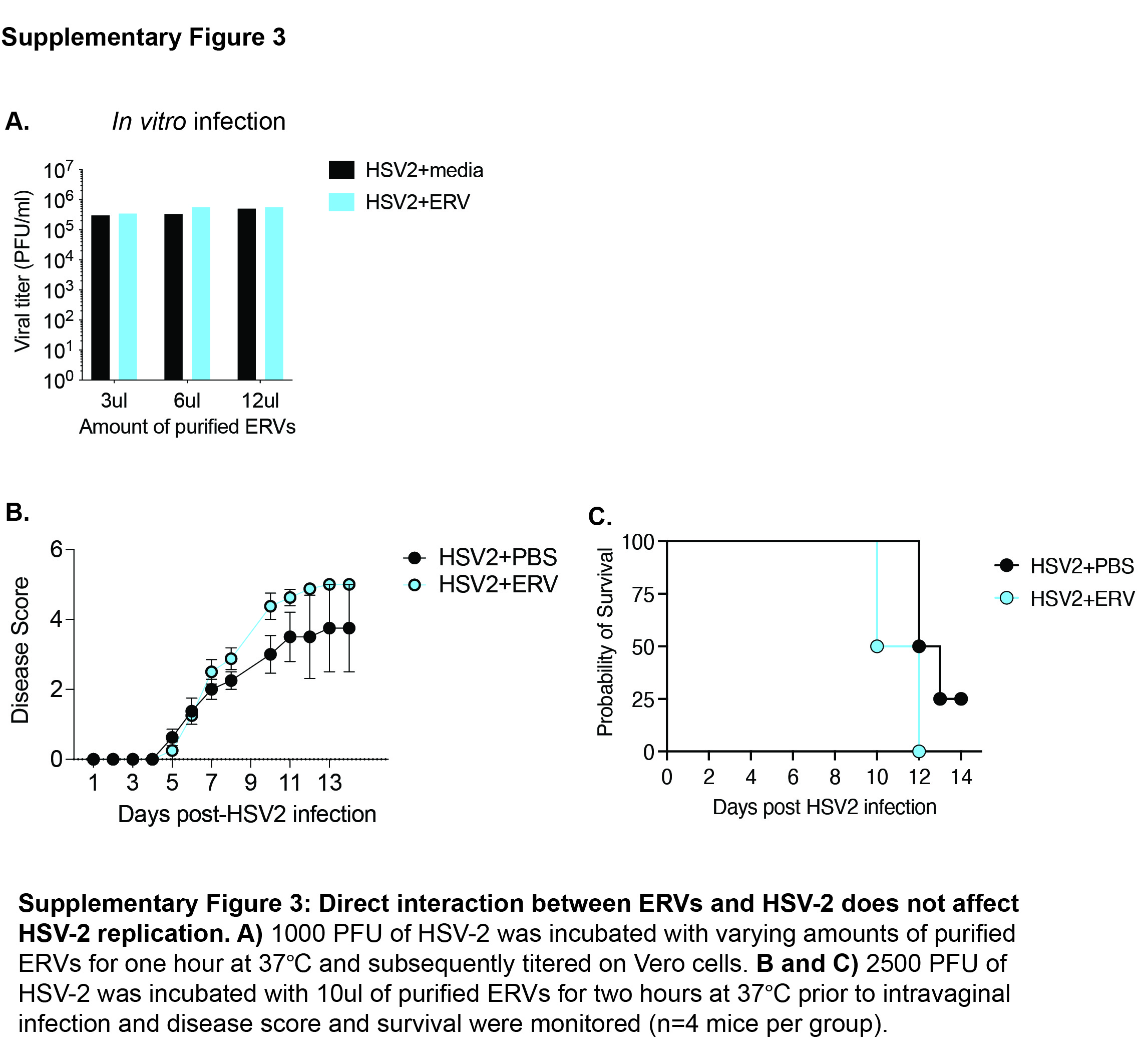

Supplement: Supplementary file 3 [file Image_3.jpeg]

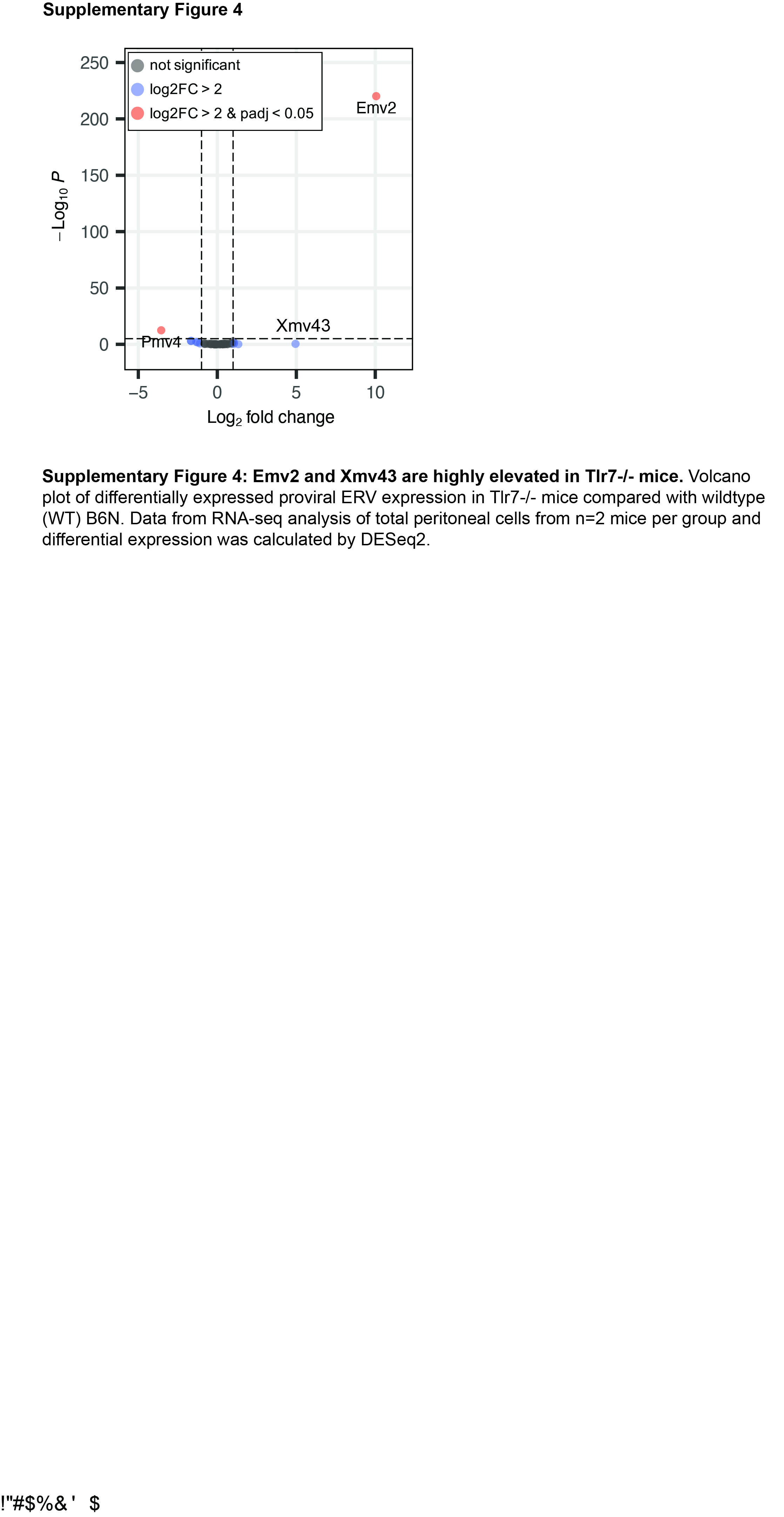

Supplement: Supplementary file 4 [file Image_4.jpeg]

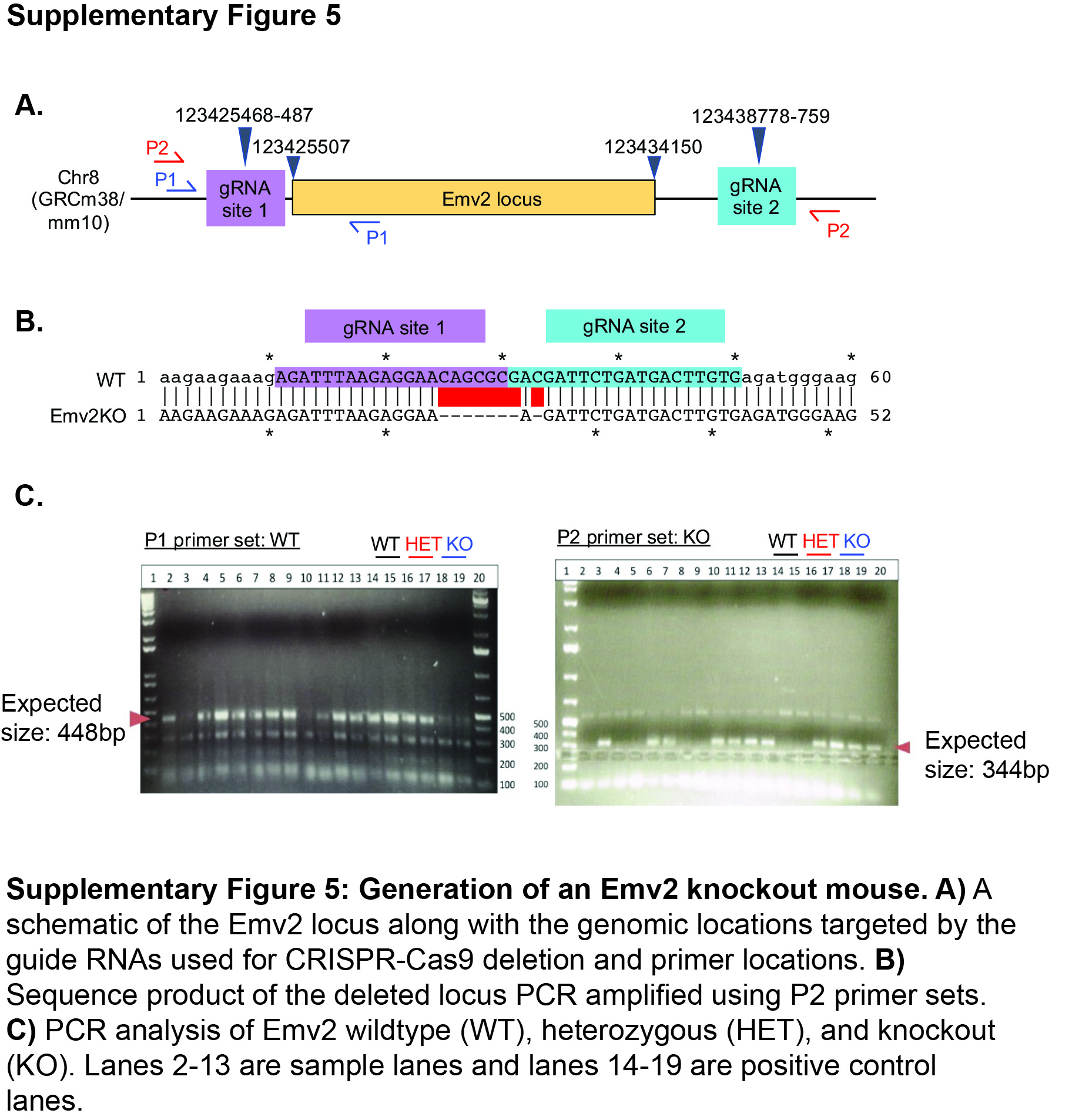

Supplement: Supplementary file 5 [file Image_5.jpeg]

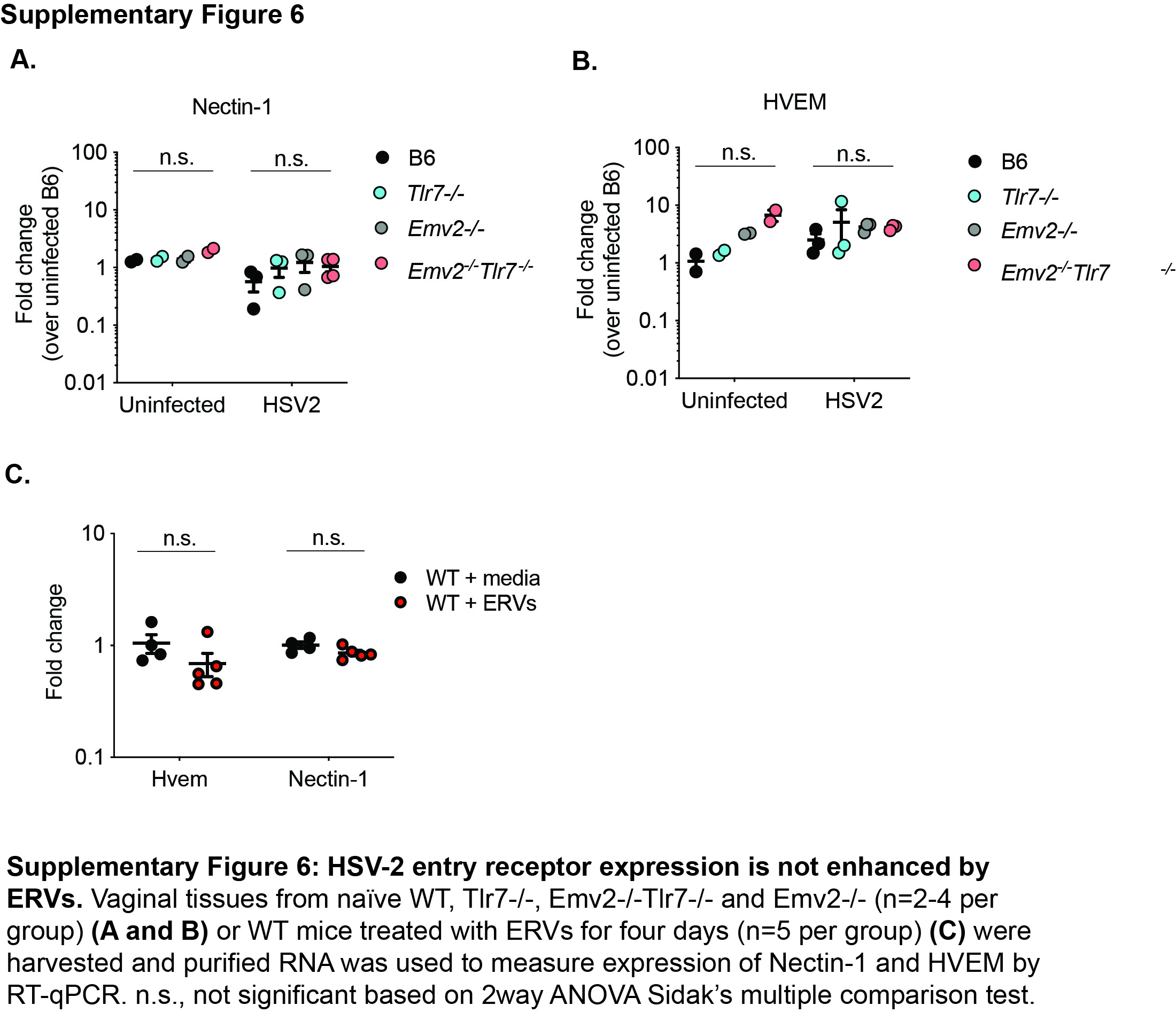

Supplement: Supplementary file 6 [file Image_6.jpeg]

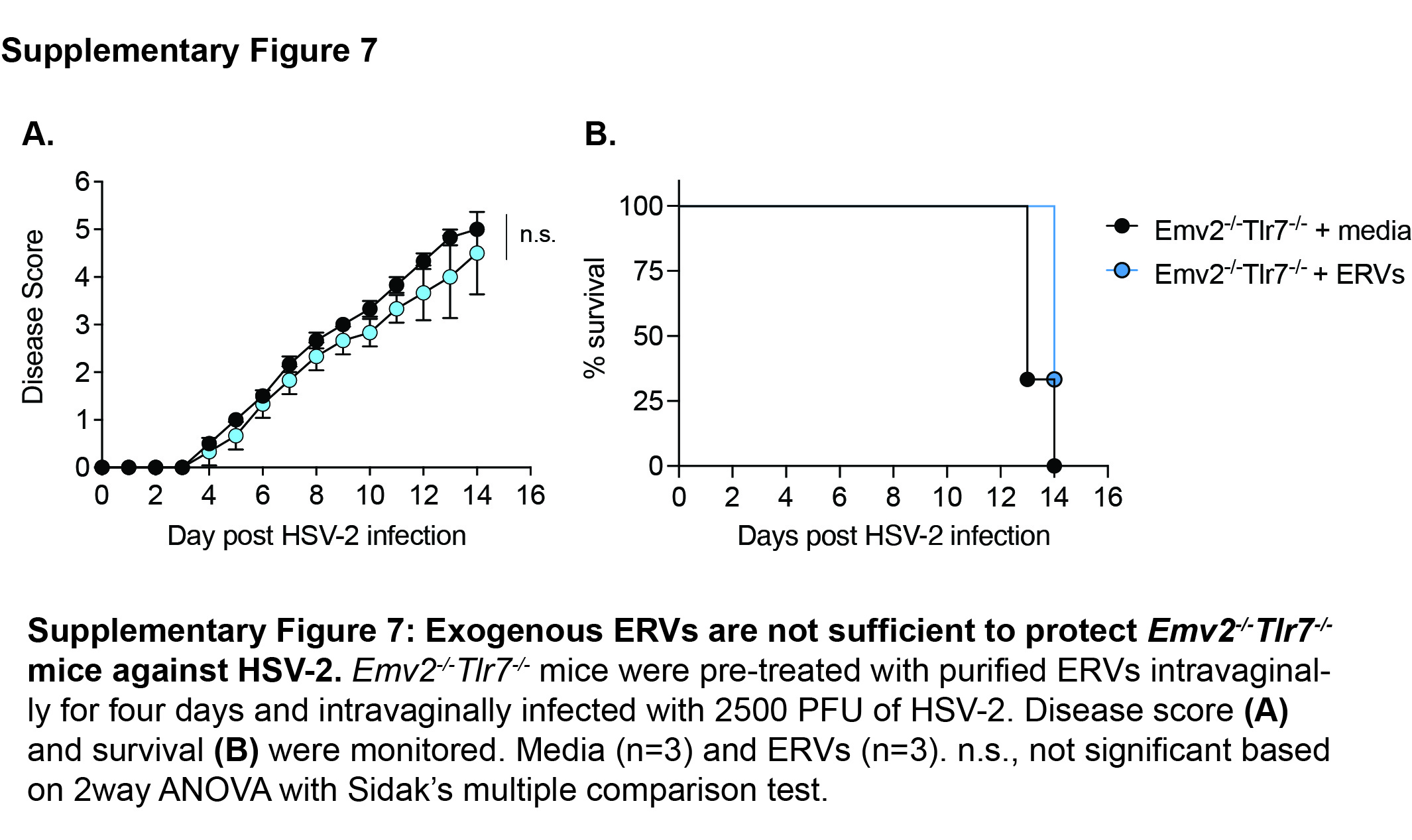

Supplement: Supplementary file 7 [file Image_7.jpeg]

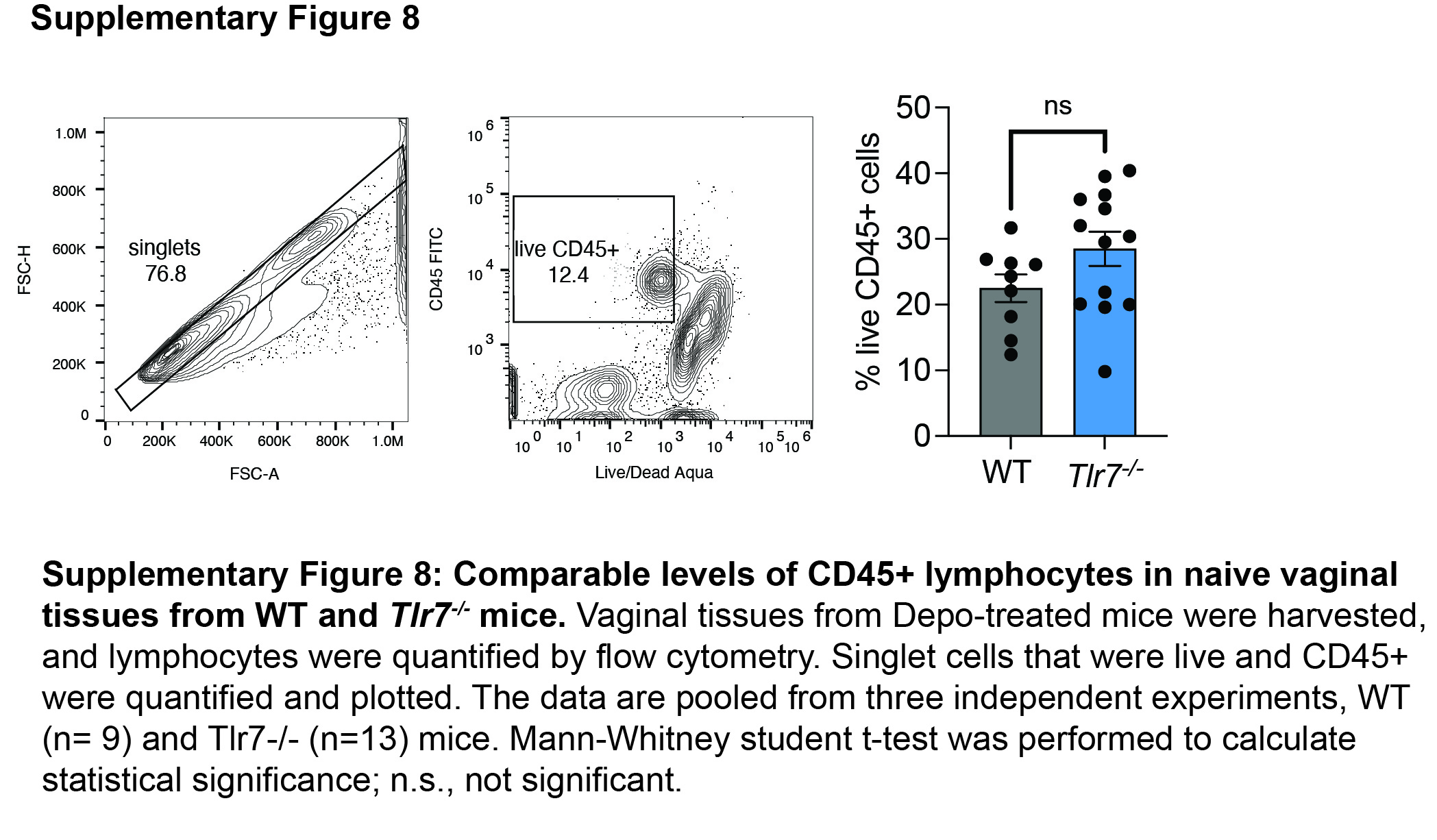

Supplement: Supplementary file 8 [file Image_8.jpeg]
